# On the effects of L2 perception and of individual differences in L1 production on L2 pronunciation

**DOI:** 10.3389/fpsyg.2014.01246

**Published:** 2014-11-05

**Authors:** Natalia Kartushina, Ulrich H. Frauenfelder

**Affiliations:** Laboratory of Experimental Psycholinguistics, Faculty of Psychology and Educational Sciences, University of GenevaGeneva, Switzerland

**Keywords:** L2 perception, L2 production, transfer, L1 production, variability in production, L2 phonology

## Abstract

The speech of late second language (L2) learners is generally marked by an accent. The dominant theoretical perspective attributes accents to deficient L2 perception arising from a transfer of L1 phonology, which is thought to influence L2 perception and production. In this study we evaluate the explanatory role of L2 perception in L2 production and explore alternative explanations arising from the L1 phonological system, such as for example, the role of L1 production. Specifically we examine the role of an individual’s L1 productions in the production of L2 vowel contrasts. Fourteen Spanish adolescents studying French at school were assessed on their perception and production of the mid-close/mid-open contrasts, /ø-œ/ and /e-ε/, which are, respectively, acoustically distinct from Spanish sounds, or similar to them. The participants’ native productions were explored to assess (1) the variability in the production of native vowels (i.e., the compactness of vowel categories in F1/F2 acoustic space), and (2) the position of the vowels in the acoustic space. The results revealed that although poorly perceived contrasts were generally produced poorly, there was no correlation between individual performance in perception and production, and no effect of L2 perception on L2 production in mixed-effects regression analyses. This result is consistent with a growing body of psycholinguistic and neuroimaging research that suggest partial dissociations between L2 perception and production. In contrast, individual differences in the compactness and position of native vowels predicted L2 production accuracy. These results point to existence of surface transfer of individual L1 phonetic realizations to L2 space and demonstrate that pre-existing features of the native space in production partly determine how new sounds can be accommodated in that space.

## INTRODUCTION

Learning a foreign language in adulthood (and often much earlier) is generally associated with difficulties in producing sounds of this language (Goto, 1971; Long, 1990; Flege, 1999; Piske et al., 2001; but see also Neufeld, 1979; Bongaerts, 1999; Gallardo del Puerto et al., 2005 for contradictory evidence showing accent-free late L2 productions). This phenomenon is commonly called having a “foreign” accent and may be described as “… phonological cues, either segmental or suprasegmental, which identify the speaker as a non-native user of the language” (Scovel, 1969, p. 38). For example, native (L1) Japanese speakers are easily identified by their accent when producing English /r/-/l/ sounds as Japanese-like /r/ (Goto, 1971; Aoyama et al., 2004). Different explanations, ranging from maturational (Lenneberg, 1967; McLaughlin, 1977; see Hyltenstam and Abrahamsson, 2008 for an overview), sociolinguistic (Lewandowski et al., 2009), and psychological (Rota and Reiterer, 2009) factors to personality-related issues (e.g., extraversion, self-esteem, and risk taking) have been proposed to account for the common phenomenon of foreign accent (see Piske et al., 2001; Dogil and Reiterer, 2009 for reviews of factors influencing L2 performance).

**FIGURE 1:**
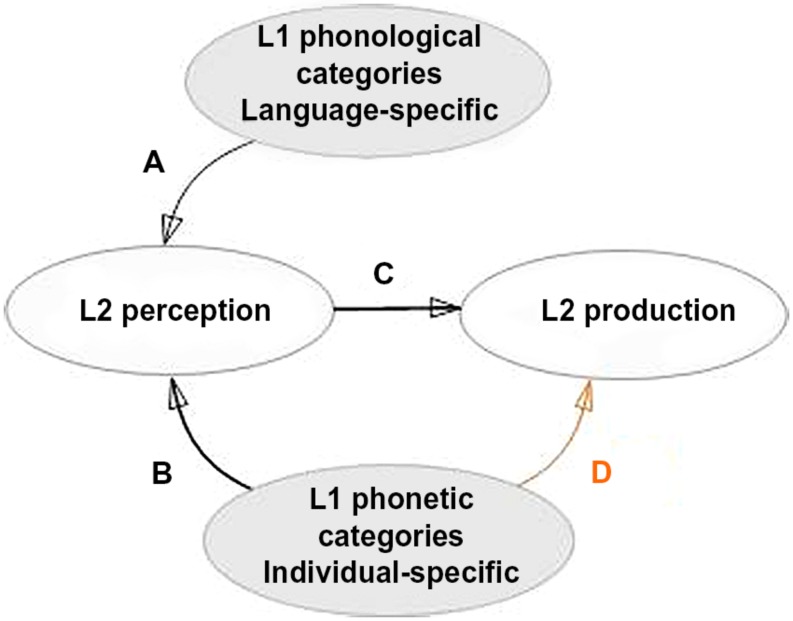
**Schematic representation of language-specific and individual-specific links between L1 phonology, L2 perception, and L2 production**.

The dominant psycholinguistic perspective (Best, 1995; Flege, 1995) attributes foreign accents to deficient L2 perception which prevents speakers from producing with native accents. Here L1 categories are assumed to transfer to L2 and to be used to perceive and then produce L2 sounds as a function of their similarity to L2 sounds. **Figure 1** presents a schematic and simplified picture^1^ of the relations between L1 and L2 perception and production. More specifically it illustrates, via links A and B, respectively, two levels of L1 to L2 transfer: abstract and surface transfer (see Major, 2008 for discussion^2^). Abstract transfer can be conceptualized as transfer of abstract L1 phonological categories/features and of perception grammar (i.e., mappings between phonemes and auditory events, Archibald, 1998; Escudero and Boersma, 2004). Studies showing the effects of L1 orthography on L2 perception, for example, are generally taken to favor abstract transfer (see Bassetti, 2008 for discussion) since orthography is an abstract representation. Surface transfer can be seen as transfer of surface phonetic properties/categories (link B). Flege’s (1995, p. 239) Speech Learning Model (SLM) model clearly defends this type of transfer, “Sounds in the L1 and L2 are related perceptually to one another at a position-sensitive allophonic level, rather than at a more abstract phonemic level”. We have shown that individual-specific phonetic properties of native productions (i.e., the position of the individual’s native category in the acoustic space and its compactness) predicted perception of L2 vowels across speakers (Kartushina and Frauenfelder, 2013). Finally, some studies suggest that transfer occurs at both abstract and surface levels (Hallé et al., 1999; Gass and Selinker, 2001). The categories established in L2 perception are assumed to be used in L2 production (link C). Flege (1995, p. 238) defends this assumption in his SLM, “without accurate perceptual ‘targets’ to guide the sensorimotor learning of L2 sounds, production of the L2 sounds will be inaccurate”. In the current study we examine links A and C, but we also explore link D, the role of individual-specific phonetic realizations in L2 production which in comparison to former two links has not received its due attention.

Both surface and abstract transfer accounts (link A and B) agree that L2 sounds are processed as a function of their perceived similarity to the transferred L1 categories. Depending upon this similarity, L2 sounds are either perceptually assimilated to native sounds, that is, integrated into an existing L1 category, or not [Perceptual Assimilation Model (PAM for naïve listeners and PAM-L2 for L2 learners), Best, 1995; Best and Tyler, 2007; SLM, Flege, 1995; Second Language Linguistic Perception (L2LP), Escudero, 2005]. L2 sounds that are perceptually assimilated to native categories are predicted to be more difficult to acquire than perceptually dissimilar (new) ones. For instance, Japanese speakers identify the English /r/ better than the English /l/ (92% versus 77% of correct responses), since the former is perceptually more distinct from the Japanese /r/ than the latter (Flege et al., 1996).

Assimilation of L2 sounds to L1 categories is assumed to take place not only in the perception of individual L2 sounds but also in the perception of L2 contrasts. According to the framework of Best (1995); Best and Tyler (2007), an L2 contrast whose two sounds map onto two different L1 categories [two category (TC) assimilation type] is predicted to be acquired better than an L2 contrast whose L2 sounds map onto one L1 category [single category (SC) or category goodness (CG) assimilation types]. L2 contrasts that assimilate in a SC manner (both L2 sounds are equally acceptable examples of an L1 category) are the most difficult to acquire. For instance, American English (AE) learners of French TC assimilate the French vowels /y/-/œ/ to the AE /u/-/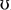/ vowels, and SC assimilate the Norwegian /y/-/i/ vowels to the AE vowel /i/. The former contrast is discriminated very well, whereas the latter is only discriminated moderately (Best et al., 1996, 2003). Finally, when both contrasting L2 sounds do not map onto any L1 category, they do not assimilate to L1; they are called uncategorized and are discriminated as a function of their similarity to each other.

The L2 category representations (established in perception) are claimed to be used in L2 production (link C in **Figure 1**; Best, 1995; Flege, 1995). For example, those Korean speakers of English who fail to perceive the difference between the English /e/ and /æ/ vowels also fail to produce them accurately. Acoustic analyses of their productions reveal that their formant spaces largely overlap (Ingram and Park, 1997). Similarly, the Japanese English learners who identify English /r/ better than /l/ improve their production of the former consonant more after 1 year of studying English (Aoyama et al., 2004; for other studies on the relationship between L2 perception and production see also Llisterri, 1995; Rochet, 1995; Flege et al., 1997; Sebastián-Gallés and Baus, 2005; Hattori and Iverson, 2010).

There are other studies, however, showing (1) no correlation (Hattori and Iverson, 2010; Peperkamp and Bouchon, 2011) or only weak correlations between L2 perception and production performance (Flege et al., 1999; Levy and Law, 2010); (2) accurate L2 production despite poor perception (Neufeld, 1979; Sheldon and Strange, 1982; Kluge et al., 2007; Kassaian, 2011); and (3) only weak or no transfer of L2 perception training to L2 production (Bradlow et al., 1997; Lopez-Soto and Kewley-Port, 2009). The lack of converging results across different L2 perception-production studies could partially be due to the differences in the methods used (e.g., tasks, stimuli) and analyses applied (e.g., comparison of average performance in perception and production of a group of speakers versus correlation analysis across individuals). Nevertheless, taken together they point to the absence of a robust relationship between L2 perception and production.

In the current study we examine the links A, C, and D (presented in **Figure 1**) in Spanish adolescents learning French at school. The Spanish phonological system contains five oral monophthongal vowels (the three “point” vowels /i/, /a/, /u/ that define the three extremes, and two middle vowels /e/, /o/) that are common to many languages, including French (Maddieson, 1984). The French vowel system (for oral vowels) is composed of 10 monophthongal vowels, six of which form three height (i.e. mid-close/mid-open) contrastive pairs (Vaissière, 2006). Importantly, Spanish lacks such height-contrastive distinctions and front rounded vowels. This allows us to test the perception and production by Spanish learners of two French mid-close/mid-open height vowel contrasts, one of which assimilates to Spanish and one that does not (the front unrounded /e/-/ε/ and the rounded /ø/-/œ/ uncategorized contrast respectively). We chose vowels over consonants for several reasons. First, vowels are more frequent and acoustically salient (e.g., they are more sonorant and longer) than consonants. Second, French has many vowel minimal pair words that are known to be of particular difficulty for L2 late speakers. Moreover, mispronunciation of the vowel height contrasts may lead to difficulties in comprehension (e.g., vowels /e/-/ε/ mark different tenses, “était”[etε]-“été” [ete] for past imperfect and past composed). Finally, although the acquisition of French vowel contrasts is one of the most difficult aspects of French phonology for L2 speakers, only few studies have explored their acquisition by Spanish speakers.

Firstly, we assessed the role of the perceptual similarity of L2 contrasts to native phonological categories during L2 perception (abstract transfer account, link A). We used five-forced-choice identification (5FCI) task. This task, unlike an ABX or two-forced-choice identification task, does not limit responses to the two members of a L2 contrast and thus, makes it possible to assess the phonological perception of L2 sounds more broadly (i.e., obtain a confusion matrix). Isolated vowels were used, due to known consonant context effects on the perception of French front rounded vowels by L2 late speakers (Levy and Strange, 2008; Levy, 2009). Our predictions about the perception of the two contrasts follow those of PAM (Best, 1995). The perception of the front unrounded French vowel contrast /e/-/ε/ is predicted to vary from poor to moderate, depending on whether both vowels are perceived as being equally good examples (SC) of the Spanish /e/ or not (CG) respectively. The second contrast, the front rounded vowels /ø/-/œ/, is of particular interest since Spanish lacks both the mid-close/mid-open vowel height distinction, and it lacks front rounded vowels (Spanish has only back rounded vowels). Therefore, according to the PAM, this contrast will be uncategorized and its perception accuracy will vary from poor to excellent depending on the perceived proximity of the /ø/-/œ/ vowels to each other. The /e/-/ε/ and /ø/-/œ/ contrasts will be referred to as assimilated (or similar) and uncategorized (or new) L2 contrasts, respectively.

Secondly, we explored the relationship between perception and production (link C) of French contrasts; more specifically, we tested the hypothesis that L2 production performance is guided by L2 perception accuracy. In order to do so we (1) compared group and individual performance in L2 perception and production (i.e., with correlations), and (2) assessed the role of L2 perception in predicting L2 production in by-subject mixed-effects regression analyses. It should be noted, that only few studies have used multiple analyses in testing L2 perception-production relationship (Flege et al., 1999; Peperkamp and Bouchon, 2011) and, to our knowledge, none have applied linear mixed-effects regression analyses that take in account within and between-speaker variability.

In order to explore this relationship we also assessed the production of /e/-/ε/ and /ø/-/œ/ contrasts. Two L2 production tasks, repetition and vowel naming, were used in order to tap into the acoustico-phonetic and phonological representations underlying L2 production, respectively. Repetition involves the activation of the auditory-motor loop: the phonological (sensory) auditory pattern is kept in working memory and transferred to the articulators. Importantly, people tend to unconsciously imitate the acoustic patterns that are phonologically irrelevant when asked to repeat (Goldinger, 1998). Therefore, we expect performance in this task to be less biased by native Spanish phonology since Spanish participants will imitate the phonetic details of French vowels. Vowel naming, on the other hand, involves no sensory input, but requires participants to recover internal L2 phonological representations which are more likely to be L1 biased. Vowel naming rather than picture naming was chosen to avoid the effect of the phonetic environment on L2-sound production accuracy (Steinlen, 2005; Baumotte, 2009; Levy and Law, 2010). Though this is an unusual task, it allows to assess phonological (context independent) representations for L2 vowels. We expect that the production of both contrasts will be less accurate in the naming than in the repetition task.

Thirdly and crucially, we looked for evidence in favor of surface transfer in L2 production by exploring the role of individual-specific L1 phonetic categories (i.e., productions of individuals in their native languages) in the production of two L2-French vowel contrasts (link D). If L2 is indeed relatively independent from L2 perception, we need to find another mechanism to explain why L2 productions are marked by a recognizable L1 accent. Native productions are highly variable between and within-speakers. Between-speaker variability refers to the position of native categories in F1/F2 space (e.g., some L1-Spanish speakers, for example, produce the Spanish /e/ vowel with a more closed mouth, whereas others produce it with a more open mouth) and within-speaker variability to the variability in production of the same sound by the same speaker (i.e., acoustic compactness of native vowel categories). Is it possible that variability in accents across L2 speakers is partly due to variability in production of native sounds (individual use of L1 speech)?

Individual differences in the position and compactness of native vowel category in L1 production have been shown to predict perception performance on similar to this category L2 sounds. Spanish speakers whose individual /e/ category was closer to the French /e/ category (reflected by the acoustic distance between the two sounds), identified it better than those whose L1 category was farther from it (Kartushina and Frauenfelder, 2013). Speakers whose native productions of the /e/ vowel were less variable (i.e., those who had a more compact vowel category) identified the L2-French /ε/ vowel better. These results support the surface transfer hypothesis and suggest that native individual-specific phonetic categories (i.e., realizations) transfer to the L2 space and influence L2 perception (link B in **Figure 1**).

Cross-language compactness of productions in L1 acoustic space has been shown to have an impact on the perception of foreign (in naïve listeners) sounds (Meunier et al., 2003). Meunier et al. (2003) found that French speakers categorized foreign (Spanish) vowels better than American speakers did. These differences were attributed to the degree of compactness of the vocalic spaces of these two languages: English vocalic space is less compact, and its vowel categories overlap more than those of French (note that both languages have 10 oral vowels in their respective inventories).

In order to test for the existence of surface transfer in L2 production (link D) we recorded the Spanish productions of the participants and analyzed them in terms of two properties of individual phonetic realizations: position of the /e/ vowel category in F1/F2 space and the compactness of vowel categories. The position of the /e/ vowel category in F1/F2 space was used to calculate the distance along the F1 and F2 dimensions between this native (individual) vowel category /e/ and each of the native target French vowel spaces (i.e., /e/ and /ε/ in the assimilated contrast) derived from recordings of native French speakers. We used Mahalanobis distance metric measure [distance score (DS)] that estimates distance between a point and a distribution. Therefore, it takes into account the natural variability of the target vowel space. This measure was also used to assess L2 production accuracy. The Mahalanobis distance has been previously used in techniques of speaker identification, where an unknown speech sample is assigned to a speaker on the basis of the minimum distance between a test speech sample and the reference samples (Majewski et al., 1979). We predicted (in line with SLM) that the closer the native category is to a similar L2 vowel, the more likely it is that the former will be used to produce the L2 vowel: the smaller the DS between the L1 and L2 vowels, the better this L2 vowel production accuracy is.

**FIGURE 2:**
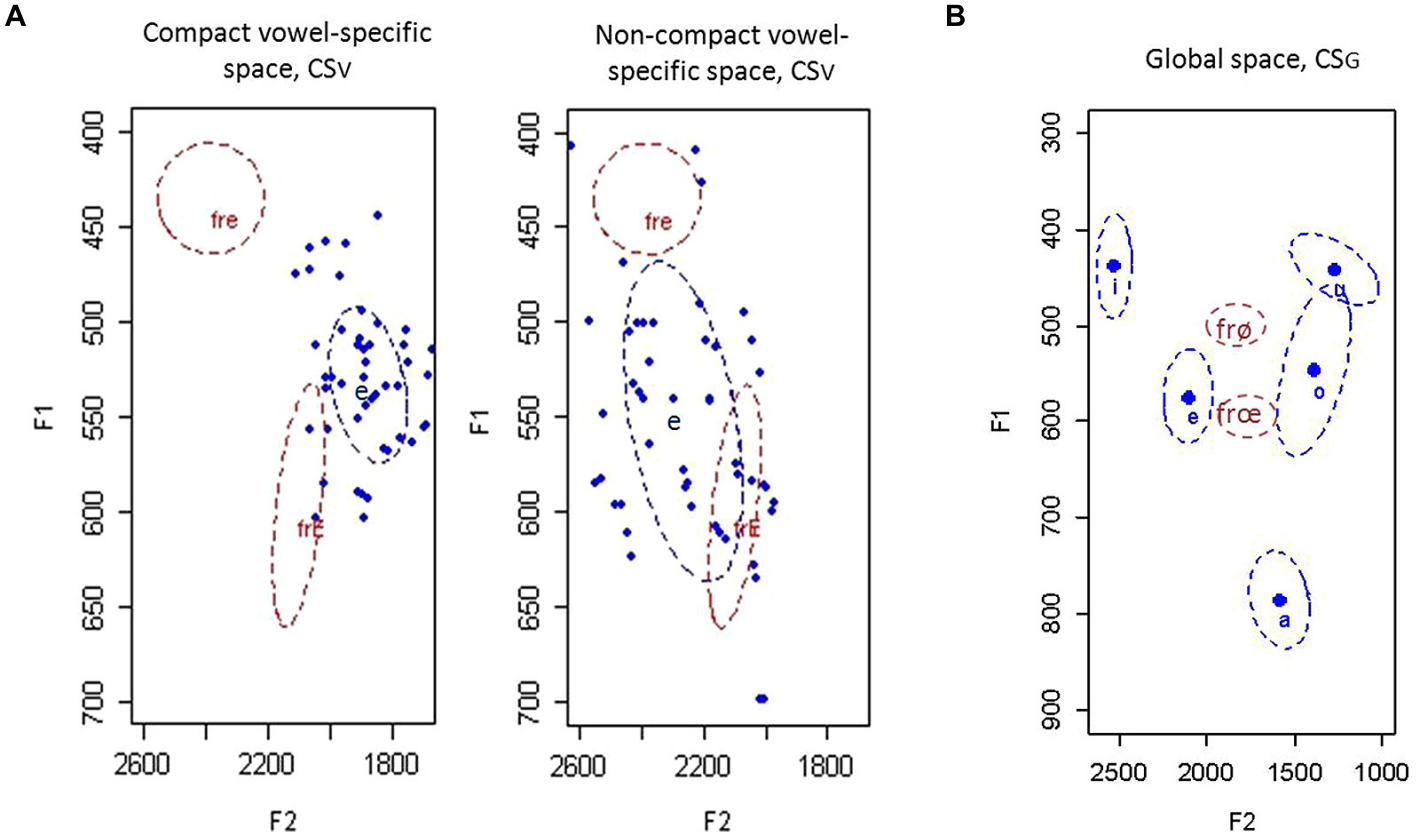
**Illustrations of Spanish L1 productions (in blue) used to compute the vowel-specific **(A)** and global **(B)** compactness scores (CS_**V**_ and CS_**G**_ respectively) along with the productions of the French target contrasts /e/-/ε/ and /ø/-/œ/ by native French speakers (in red).** Ellipses reflect 1 standard deviation from the mean formant values F1 and F2. **(A)** The blue ellipses represent compact and non-compact acoustic spaces for the Spanish /e/ vowel displayed with the red ellipses for the French /e/ (fre) and /ε/ (frE) vowels. Dots represent 25 individual productions of two participants for the Spanish /e/ vowel. **(B)** The five acoustic spaces (in blue) for Spanish vowels /i, e, a, o, u/ were used in computing the global compactness score. They are compared to the acoustic spaces for the French vowels /ø/ and /œ/ (fre and frE respectively).

The second property of native individual productions, the compactness of vowel categories, was measured in two ways: (1) a vowel-specific compactness score (CS_V_) corresponding to the variability of the /e/ vowel in the L1 acoustic space, and (2) a global compactness score (CS_G_) corresponding to the sum of the five CS_V_ for the five Spanish vowels [see **Figure 2** for an illustration of the two compactness scores (CSs)]. The CS_V_, for example, was calculated as the area of an ellipse (the distribution of the produced tokens in F1/F2 space was assumed to be elliptical) having major and minor axes with a length of 1 standard deviation of the mean along the given axis.

The CS_V_ was used to predict the production performance in the assimilated French contrast. Difficulties in the production of L2 assimilated contrasts are generally attributed to the use of L1 categories to which they assimilate (e.g., the English /i/ and /I/ vowels are produced as one Spanish /i/ vowel, Morrison, 2006; see Flege, 1995 for other examples). Since the CS provides a measure of the size of the L2 vowel category, we expected that the likelihood that an L2 sound will be close to an L1 category within the interphonological space decreases with the compactness of this L1 category: the more compact the L1 vowel category /e/ is, the more accurate the productions of similar L2 vowels will be in both naming and repetition tasks.

The CS_G_ was used to predict the production accuracy of the uncategorized L2 vowels. The CS_G_ is taken as a global measure of within-category variability in the acoustic space. We expected that speakers whose within-category variability is smaller (a compact acoustic space) would have larger between-category spaces, i.e., larger available slots. It captures, therefore, indirectly the size of available acoustic slots in native space. Vowels were checked by eye for each speaker for evenness of their distribution in the acoustic space. Like the SLM, we expect that: (1) the creation of a new category is easier when the L2 sound falls within an empty region of the L1 phonological space, i.e., this is what defines a new sound in the SLM; and (2) the L1 and L2 phonetic categories exist in one phonological space, and are related to one another at a position-sensitive allophonic level (Flege, 1995; also Escudero and Boersma, 2004). The likelihood that a new L2 sound falls within or close to the borders of L1 phonemes is greater in a non-compact acoustic space compared to a compact one. Therefore, we expected that speakers with smaller CS_G_ will have higher production accuracy on the /ø/-/œ/ vowels.

The present study firstly reports the results of an analysis of the perception and production of L2 French contrasts. Secondly, it reports the results of the acoustic analyses of vowel productions in Spanish. Finally, it examines the role of L2 perception and L1 production in L2 production.

## MATERIALS AND METHODS

### PARTICIPANTS

Fourteen monolingual native female speakers of Spanish (mean age 16 years) from a Spanish middle school in Plasencia (Spain) took part in the study. They had, on average, 4 years of French classes at school and, according to their teacher, their proficiency in French corresponded to the intermediate B1 level of the Common European Framework of reference for languages. They all came from the same region in Spain and had never lived in a French-speaking country. Participants gave informed consent and were free to withdraw from the experiment at any time.

### STIMULI

To create the stimuli used to assess the production and perception performance of the Spanish participants on French vowels, seven native female French speakers were recorded. They were all from the same French region (Paris, Ile-de-France) to minimize the effect of regional dialect. The productions of multiple speakers were used to increase the variability across vowels for two reasons; first, it encourages phonological rather than acoustic perception of vowels (Lively et al., 1993) and second, it avoids the one-speaker bias (Baker and Trofimovitch, 2006).

#### Stimuli for identification and repetition tasks

Six of these seven native French speakers were recorded while reading three lists of 10 French sentences of the type, “Je prononce /ε/ comme dans lait” ([ʒə pronõs ε kom dã lε], “I pronounce /ε/ as in milk”). Each list contained 10 sentences with one of the 10 oral French vowels examined. Each sentence contained one isolated vowel and also a word containing this same vowel (e.g., the vowel /ε/ in isolation and in the word /lε/ (lait, “milk”). The sentences in the three lists were identical except that the final word of the sentence began with a different consonant in each list: /l/ (lait-/lε/ “milk”), /s/ (sait-/sε/ “know”), or /p/ (paix-/pε/ “peace”). Recordings were made in a quiet room, using a Marantz PMD670 portable recorder and a Shure Beta 58A microphone, sampled at 22.05 kHz directly to 16-bit mono.wav files.

The vowels from the words were extracted from the sentences, normalized for intensity, and matched in length (mean 210 ms). These vowels were then rated by five French speech therapists; the vowels that were accepted as good prototypes of French by at least four judges were kept. In total, 120 vowel productions were retained for the identification test^3^: 10 vowels^4^ * 6 different speakers * 2 exemplars per speaker.

Forty vowel productions were used for the repetition task; this included four isolated vowels (i.e., /e/-/ε/-/ø/-/œ/) * 5 different speakers * 2 exemplars per speaker. These 40 vowels were also used to define the acoustic spaces for the French vowels /e/-/ε/-/ø/-/œ/ (i.e., 10 tokens * 4 vowels) when computing the DS, a measure of L2 production accuracy that we elaborated to assess L2 speakers’ production performance (see Evaluation of L2 Production Accuracy for details).

In addition to sentence reading, the sixth native speaker read a list of 10 words that named the pictures in the familiarization phase of the identification task. Monosyllabic, non-cognate, concrete nouns which were likely to be known by the participants were used, each containing one of the 10 French vowels of interest.

#### Stimuli for naming task

The seventh French speaker was recorded (using the same recording parameters as above) while reading a list of other (than used in identification) 20 different words (i.e., five words for each of the four tested vowels /e/-/ε/-/ø/-/œ/) whose corresponding pictures were used in the naming task. These productions were used in the familiarization phase of the naming task. For example, the five proposed words for the vowel /ε/ were: chaise [ʃ**ε**z], chèvre [ʃ**ε**vr], lèvres [l**ε**vr], pêche [pεʃ], and tête [t**ε**t] (“chair,” “goat,” “lips,” “peach,” and “head,” respectively); pictures depicting these words were used in the task (see **Table 1**). All 20 words were monosyllabic, frequent, concrete, non-cognate French words that were likely to be known by the pupils (as confirmed by the L2 French teacher of the tested participants). Note that the phonetic contexts of each tested vowel (i.e., phonemes that preceded and followed the vowel) were varied due to known effects of phonetic context on production accuracy (Sheldon and Strange, 1982; Steinlen, 2005; Baumotte, 2009).

**Table 1:** Words and corresponding pictures used in naming task for the vowel /ε/.

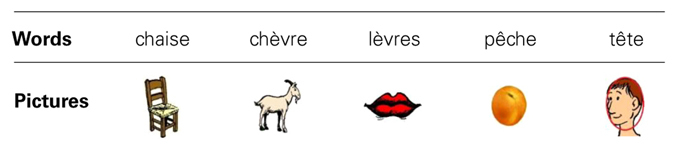

### PROCEDURE

Participants first filled in a language-background questionnaire concerning, among other things, their L1, fluency in French and their proficiency in other languages. After that, they performed three tasks with the French stimuli; their order was randomized across subjects. All participants performed the Spanish reading task last. All tasks were performed individually on a Dell laptop using E-Prime 2 software (Psychology Software Tools, Pittsburgh, PA, USA). Participants’ productions were recorded with a professional digital recorder Marantz PMD670, using a Sennheiser PC151 headphone with microphone, they were sampled at 22.05 kHz directly to 16-bit mono.wav files.

#### Perception test

A 5FCI task was used to assess the perception of L2 vowels. The 10 tested French oral vowels were separated into two groups of five vowels as function of their possible perceptual confusability with each other for Spanish speakers. Group one contained the mid-close-mid-open front unrounded (assimilated) contrast /e/-/ε/, along with the other three vowels /i/, /u/, /y/. Group two contained the mid-close-mid-open rounded front (non-assimilated) contrast /ø/-/œ/, along with the other three vowels /o/, /ɔ/ and /a/ (see **Table 2**). The perception performance on the vowels other than the /e/-/ε/ and /ø/-/œ/ vowels was tested as part of a bigger project, and the results are not reported in this study. The identification performance on each group of vowels was assessed separately using the same two-phase procedure, i.e., a familiarization and a testing phase.

**Table 2:** Words corresponding to the vowels and pictures used in the identification task.

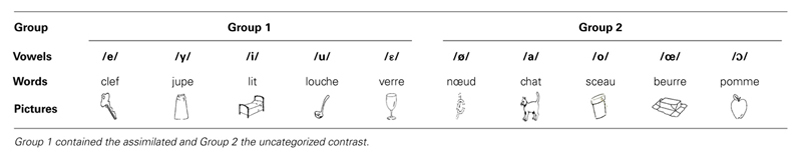

During the familiarization phase, participants had to learn the association between the pictures presented on the screen and the vowels in their labels. **Table 2** presents the words and pictures that were used to represent the vowels. The words were selected using the following criteria: they had to be monosyllabic, concrete, non-cognate and most importantly, familiar to the pupils (as confirmed by the L2 French teacher of the tested participants) and of comparable frequency (if possible). Pictures rather than letters or written words were chosen in order to avoid an orthographical response bias (Bassetti, 2008). The combination of vowel, word and picture was presented five times.

**FIGURE 3:**
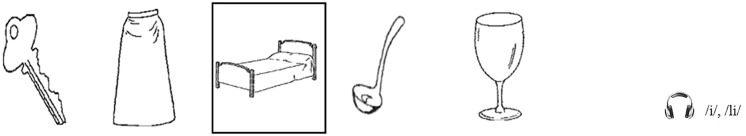
**An example of a trial for the familiarization phase.** Participants saw the picture of a bed that was indicated by a rectangular frame, and heard the vowel /i/ and the word “lit” (“bed”) via headphones.

At the beginning of the familiarization phase, the five pictures of a group appeared on the screen and remained there for the duration of the familiarization phase for that group. On each trial, one of the five pictures was indicated by a rectangular frame and the corresponding vowel and word were heard (see **Figure 3** for an example of a trial). The order of the indicated pictures was fixed from left to right. There were five trials per vowel. The vowels and words produced by one of the native French speakers (i.e., the sixth speaker) were used. The familiarization phase was immediately followed by the testing phase.

On each trial of the testing phase, the participants heard one of the five isolated vowels (recorded from the other five speakers, i.e., from 1 to 5^5^), and were presented with five pictures that they had been familiarized with earlier (see **Table 2** and **Figure 3**). They were asked to click with a mouse on the picture that corresponded to the vowel heard. No time limit was imposed for answering. No feedback on their performance was given. Each of the two target vowels was presented 10 times in random order along with the three other vowels included in the group. The same procedure (i.e., familiarization and testing) was used to assess identification performance on the other group of vowels.

#### Spanish productions

To test the participants’ L1 production, a reading task was used. The participants read a passage from a chapter of a SP translation of “The Godson” by Leo Tolstoy. They were instructed to read as naturally as possible at a moderate tempo. This task, rather than natural speech was used in order to control the phonetic environment of the produced vowels across the participants. The acoustic values of the five vowels (i.e., /i/, /e/, /a/, /o/, /u/) produced in Spanish were used to compute two measures of L1 compactness: a CS_V_ and a CS_G._ They were also used to calculate the Mahalanobis DS measure between the native vowel category /e/ and French /e/ and /ε/ categories.

***L1 compactness scores.*** A CS was computed for each participant and for each of the five SP vowels using a formula which was derived from the following mathematical formula used to calculate the area of an ellipse:

A⁢r⁢e⁢a=a⁢b⁢π

where a and b are 1/2 the length of the ellipse’s major and minor axes. Since the distribution of the productions in F1/F2 space was assumed to be elliptical, the angles of the major and minor axes of an ellipse centered on the mean of the productions were estimated (in order to determine the orientation of the axes). The formula used to calculate the CS was:

C⁢S=σ⁢F⁢1⁢σ⁢F⁢2⁢π

where *σ_F1_* is 1 standard deviation of the mean of F1, and *σ_F2_* is 1 standard deviation of the mean of F2.

A CS was computed for each participant and for each vowel. The CS for the SP /e/ vowel is called CS_V_. Before computing the CS_G_, speakers’ mean F1 and F2 values for five Spanish vowels were checked by eye for the evenness of their distributions in the acoustic space, and that for each speaker. This revealed that all vowels were produced evenly occupying the five corners of Spanish vocalic space; there were no superimposed vowels. By this analysis, we can eliminate the possibility of there being a very compact space but no room for new sounds. A CS_G_ was computed for each participant by taking the sum of the five CS, of the five SP vowels. The CS_V_ and CS_G_ were used as predictors in mixed-effects models analyses of the production of the French (FR) assimilated (i.e., /e/-/ε/) and uncategorized (i.e., /ø/-/œ/) contrasts respectively.

***Distance score from native Spanish /e/ to the non-native French /e/ and /ε/ vowels.*** Mahalanobis DS was used to compute the distance between the native Spanish /e/ and the French /e/ and /ε/ vowels (DS-FR). For each speaker and for every exemplar of the native vowel /e/, two DSs from this exemplar were computed to the native French target spaces for the /e/ and /ε/ vowels, respectively. The latter were derived from the productions of the five native French speakers (see Stimuli).

#### French productions

***Repetition task.*** On each trial of the repetition task, participants first saw a cross in the middle of the screen for 500 ms and then heard an isolated French vowel (one of the four tested) via headphones. They were asked to repeat it as correctly as possible without time constraints, and their repetitions were recorded using a digital recorder. The presentation of the next trial was controlled by the participants. Five exemplars of each vowel (i.e., produced by five native French speakers) were used in this task, and each of the exemplars was repeated two times. In total, 40 stimuli were presented in randomized order.

***Naming task.*** For the naming task, participants were first familiarized with pictures, and they were then tested on these pictures.

On each trial of the familiarization phase, a visual and an auditory stimulus were presented simultaneously. The former was a picture that appeared on the screen and the latter was the spoken word (via headphones) that corresponded to that picture. The productions of the seventh speaker were used for this task. Each vowel was represented by five different words (see Stimuli for details). The words and pictures used here were different than those used in the identification task. Each picture-word combination was presented twice, resulting in 40 trials in total: five pictures * 4 vowels * 2 times.

The testing phase immediately followed the familiarization phase. On each trial, a cross appeared on the screen for 500 ms, and was then followed by a picture that was displayed for 4000 ms. Participants were instructed to produce the vowel that the word (i.e., picture) contained (e.g., for the picture of an “arrow” – “flèche” [*fl*ε*∫*] participants had to produce [***ε***]) as correctly as possible. Note that participants heard no auditory model of the target word during the testing phase. The participants were allowed to skip trials if they did not know or had forgotten the names of presented pictures. In this case, for the trial in question, they had to say “no answer” and continue to the next trial.

#### Evaluation of L2 production accuracy

To assess the accuracy of vowel productions in the naming and repetition tasks, Mahalanobis DS was computed which represented the distance between the vowel produced by participants to the corresponding FR target vowel acoustic space. The target space was derived from the productions of the 5 native French speakers (see Stimuli). The calculations were implemented in Matlab (2011a; The MathWorks Inc., Natick, MA, USA). We used this metric rather than the simpler Euclidean distance of the produced token to the mean target FR vowel in order to take into account the natural variability in speech production (i.e., in this case that of the target vowels). In addition, in order to assess the distinctness in production of the pairs of height-contrastive vowels within each contrast, an acoustic analysis (i.e., F1) of the produced French vowels by Spanish participants as compared to native French speakers was performed.

## RESULTS

### L2 PERCEPTION

To assess the effect of contrast type (assimilated [/e/-/ε/] versus uncategorized [/ø/-/œ/]) on identification performance, accuracy scores (1 for correct and 0 for incorrect) were fitted to a mixed-effects logistic model that is traditionally used to analyze binomially distributed data (Baayen, 2008; Quené and van den Bergh, 2008). Contrast type was included as fixed factor; by-participants and by-vowels random slopes, both adjusted for the correlations with the fixed factor were included as random-effects factor (i.e., resulting in a maximal random-effects structure according to Barr et al., 2013). All statistical analyses were run using the R software (R-project, R Development Core Team, 2011). There was a significant effect of contrast type (β = 0.56, SE = 0.27, *t* = 2.042, *p* < 0.05), with the assimilated vowels (51% of correct answers) being better identified than uncategorized ones (37%). A confusion matrix (**Table 3**) shows the errors (misidentification) on the perception of the /e/, /ε/, /ø/, and /œ/ vowels.

**Table 3:** Confusion matrixes with percent identification responses for the vowels /e/ and /ε/ in A and /ø/ and /œ/ in B.

Presented vowel	% Identification as					
	e	ε	u	i	y
**A**
e	59	35	5	1	0
ε	55	43	2	0	0

	ø	œ	o	ɔ	a

**B**
ø	40	33	11	14	2
œ	36.4	34.2	6	19.4	4

### L1 PRODUCTION

The material recorded in the Spanish reading task was analyzed by the first author using the Praat software (Boersma and Weenink, 2010) and following the following procedure: (1) The acoustic quality of the recordings was checked (e.g., intensity, and presence of extraneous sounds such as coughing, sneezing, and sighing). (2) The vowel segments (embedded in words) were located by ear and by eye (i.e., using the spectrograms). Default Praat settings were used to track vowel formant contours. Their quality was checked by eye for each individual. (3) If the formant contours were tracked erroneously, the maximum number of formants and the ceiling of the formant search range were changed in order to maximally approximate the formant contours and the darker (corresponding) bands on the spectrogram. (4) Vowels whose length was less than 30 ms or whose formant contours were unstable were rejected from the analyses. As a result of these steps, 6% of the data were discarded. The F1 and F2 of the remaining vowels (3568 in total) were calculated at the mid-point of the segment. (5) Finally, two compactness (CS_V_ and CS_G_) and two DS-FR (for French /e/ and /ε/) scores were computed for each L2 speaker. In total, 14 CS_V_s (mean = 259 kHz^2^, SD = 97 kHz^2^), 14 CS_G_s (mean = 1187 kHz^2^, SD = 261 kHz^2^), and 28 DS-FRs (mean = 2.54, SD = 0.53) were computed, and were used in mixed-effects models analyses as predictors of the production performance on the uncategorized and assimilated French contrasts.

### L2 PRODUCTION

The quality of the tokens produced during the L2 production tests was checked by the first author for intensity and presence of non-linguistic sounds (e.g., coughs, sneezes, sighs). As a result, 13 tokens produced in the repetition task and fifteen in the naming task were removed. In addition, 90 trials without answer (i.e., “no answer” trials) in the naming task were also discarded. The F1 and F2 values of the remaining tokens were calculated as in the previous analyses.

The DS was computed to assess the production accuracy in the L2 naming and repetition tasks between the produced tokens and the French target spaces. 847 DSs were obtained for both tasks (naming and repetition). Outliers and extreme values were detected using Quantile–Quantile (Q–Q) plots and were removed. The remaining DSs varied from 0.1 to 12.72, and had a standard deviation of 2.31. The following statistical parameters are reported: the coefficient estimate β, the standard error (SE), and the *t*-value (t).

To analyze the production of the assimilated and uncategorized FR vowels, the DSs were fitted to a general linear mixed-effects model (Baayen, 2008; Baayen et al., 2008; Barr et al., 2013). The effects of compactness, task (repetition versus naming), contrast type (assimilated versus uncategorized) and identification accuracy (1 for correct, 0 for wrong answer) were included as fixed factors; similar to the analyses on the perception data, by-subject and by-vowel random slopes with correlation parameters were included in the model as random-effects, leading to a “maximal” random-effects structure (Baayen et al., 2008; Barr et al., 2013; Winter, 2013).

**FIGURE 4:**
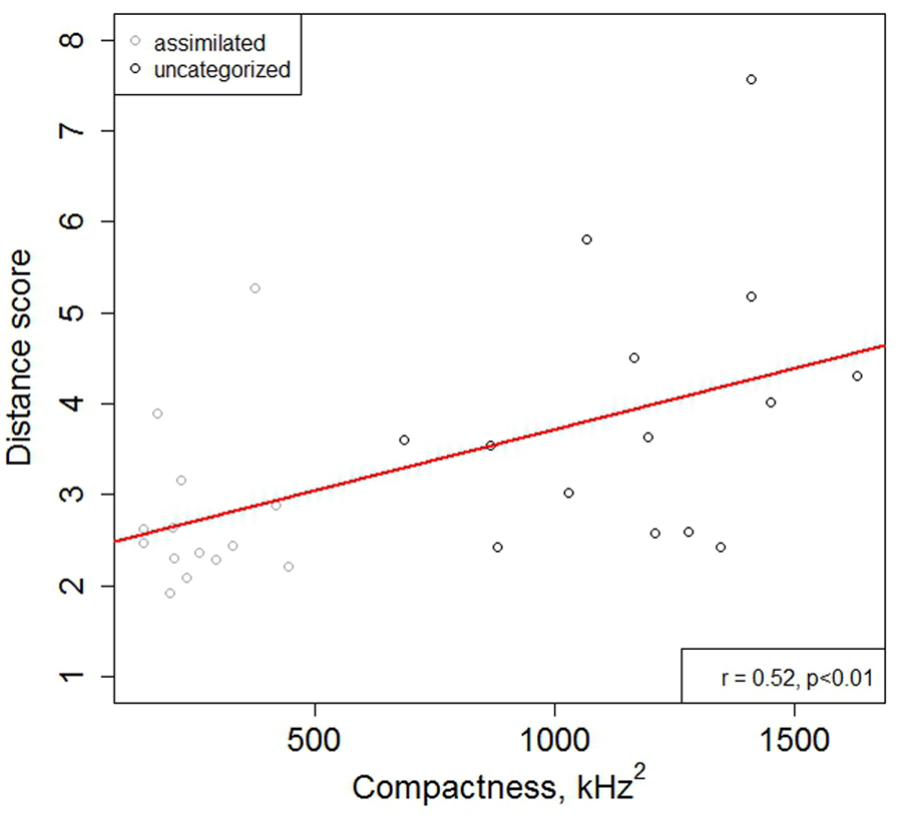
**Scatterplot of mean individual productions of the assimilated and uncategorized contrasts and regression line for the effect of L1 compactness on L2 production accuracy score [distance score (DS)]**.

Results showed that there was a significant effect of compactness (β = 2.481e-06, SE = 1.173e-06, *t* = 2.115), indicating that speakers with more compact L1 acoustic spaces produced L2 vowels better than those whose L1 acoustic spaces were more variable. There was also an effect of contrast type (β = 4.558e, SE = 1.329e, *t* = 3.429), indicating that assimilated vowels were produced better than uncategorized ones, with the DSs being closer to the FR target vowel mean in the former condition (2.75 and 3.96 DS units, respectively). There was no effect of task (*t* < 1), nor of the identification score (*t* < 1). Additional correlation analyses between L1 compactness (CS_V_ and CS_G_) and mean L2 production accuracy for the assimilated and uncategorized contrasts revealed a significant correlation (*r* = 0.52, *p* < 0.01; see **Figure 4**).

**FIGURE 5:**
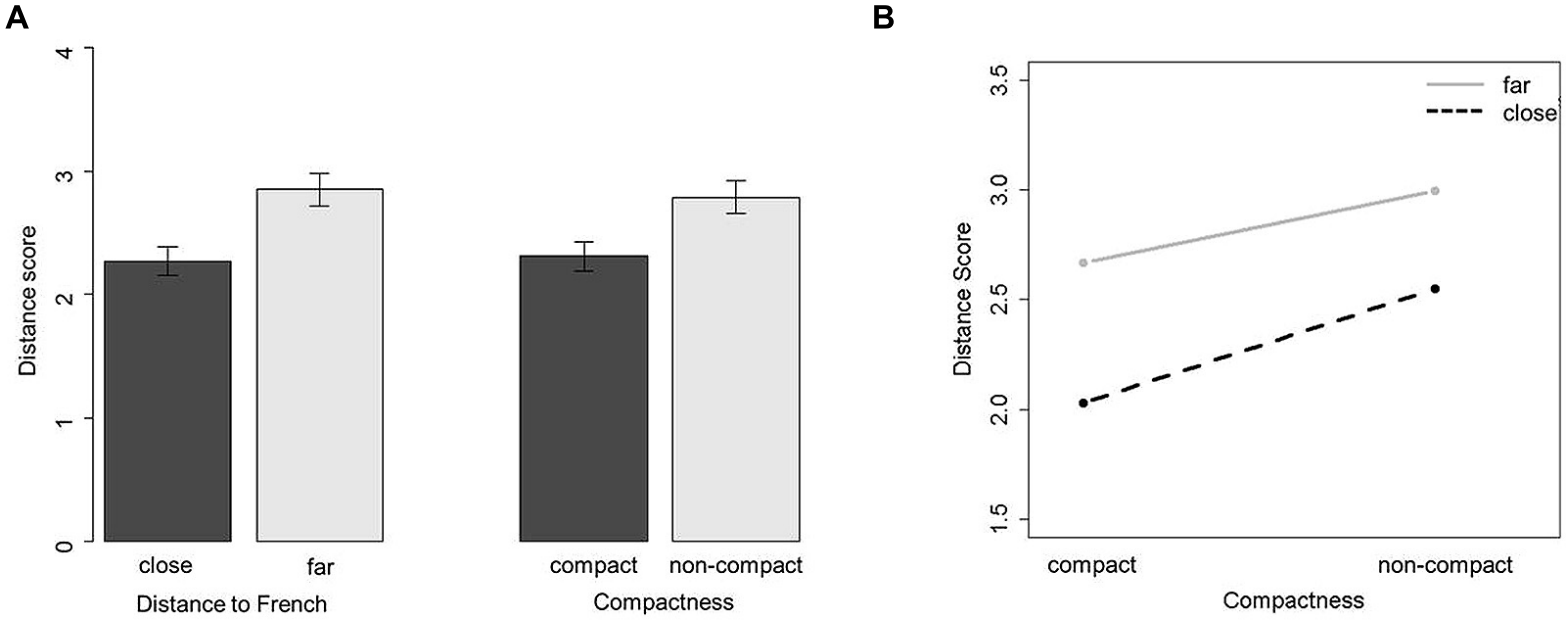
**(A)** Mean DS for two groups of speakers on the left panel whose L1 productions are close to versus far from French vowels, and on the right pane, for two groups of speakers whose L1 space is compact versus non-compact. Mean DSs are shown, and error bars represent ±1 standard error of the mean. **(B)** Mean DSs for speakers with compact and non-compact acoustic spaces for groups whose L1 productions are close to and far from French.

In order to assess the effect of the distance between the Spanish /e/ and the French /e/ and /ε/ vowels (DS-FR) on the production of the French similar vowels, separate general linear mixed-effects analyses were applied to the data set on assimilated vowels only. Similar to the previous analyses, the fixed-effects structure included the effect of compactness, task, and identification accuracy; in addition, we included the fixed effect of DS-FR and its interaction with the CS_V_. The random structure included by-subject and by-vowel slopes adjusted for the fixed factors. There was a significant effect of the DS-FR (β = 4.089, SE = 1.287, *t* = 3.176), indicating that speakers whose L1 productions of the /e/ vowel were closer to the target French vowel category produced this target more accurately than those whose L1 /e/ vowel was farther from the French target space. There was also a significant effect of compactness (β = 2.077e-05, SE = 1.070e-05, *t* = 1.941) indicating that speakers with more compact acoustic space for Spanish /e/ vowel produced French /e/ and /ε/ vowels better than those whose L1 acoustic space was more variable; and a significant interaction between the DS-FR and CS (β = -1.079e-05, SE = 5.193e-06, *t* = –2.078) indicating that the effect of compactness is stronger when the acoustic distance between the Spanish and French sounds is lower. In order to illustrate these results, Spanish participants were divided into two groups of seven speakers in each as function of the acoustic distance of their L1 /e/ productions to French vowels /e/ and /ε/: those whose L1 vowel is close and those whose L1 vowel is far. They were also divided into two groups of seven speakers as function of their compactness in L1 space: compact and distributed spaces. **Figure 5** illustrates the main effects of compactness and distance to French vowels and their interaction on L2 production accuracy. The factors of task and identification accuracy did not reach significance (*t* < 1).

**FIGURE 6:**
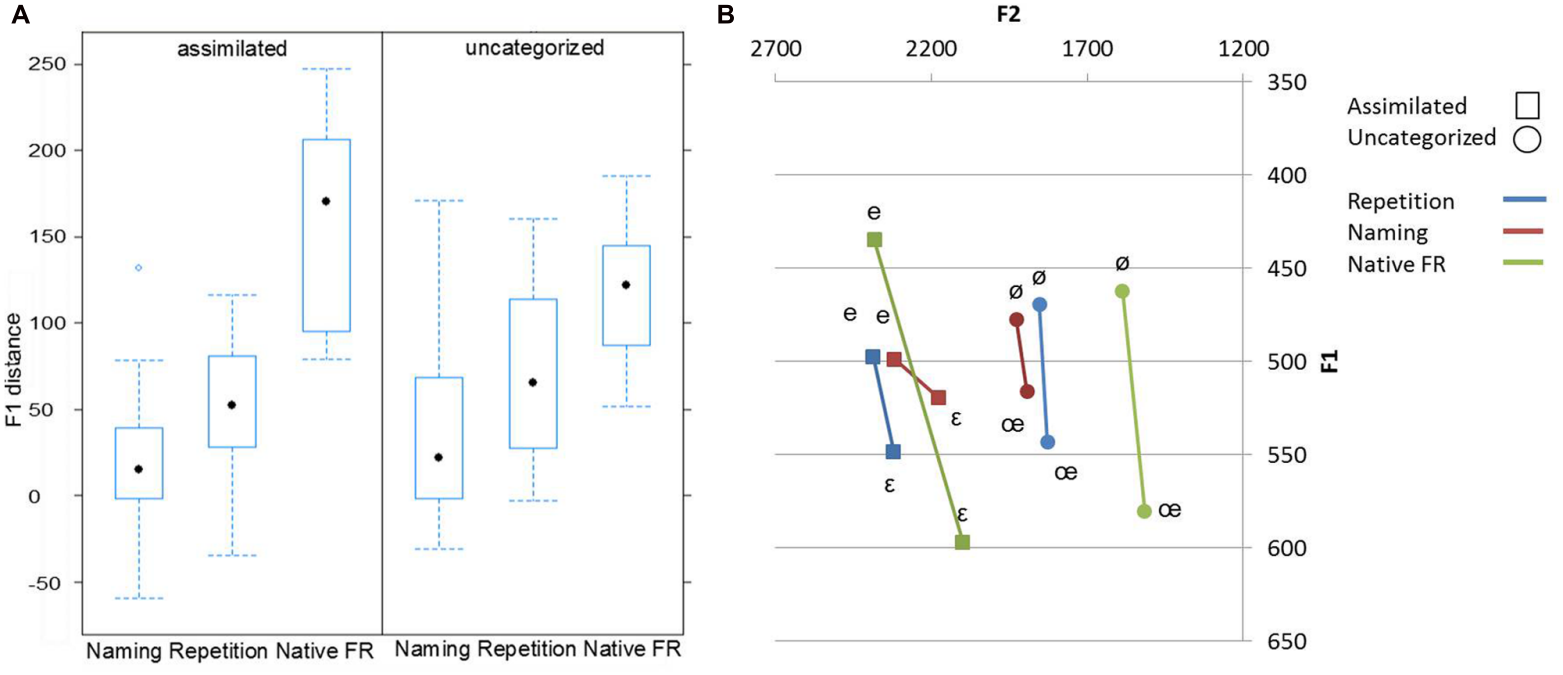
**F1-distances between the contrastive vowels produced by Spanish speakers in the naming and repetition tasks and by native French speakers. (A)** Median, lower and upper quartiles and the minimum and maximum F1-distances as function of task and contrast. **(B)** Mean F1-distances for assimilated and uncategorized contrasts as function of task.

An acoustic analysis of the produced French vowels was performed in order to examine how well L1-Spanish speakers produced the pairs of height-contrastive vowels within each contrast, compared to native French speakers. The F1 is the acoustic parameter that is the most indicative of height differences. F1-differences between the /e/–/ε/ vowels, and between the /ø/–/œ/ vowels were computed for each subject for the French vowels produced by Spanish and French speakers separately. Bartlett’s test did not show a violation of homogeneity of variances [χ^2^ (1) = 0.0834, *p* = 0.77]. An ANOVA was performed on the resulting F1 difference values, with a between-group factor of contrast (/e/ versus /ε/, and /ø/ versus /œ/), and with a within group factor of task (naming, repetition, and native productions). There was a significant effect of task [*F*(2,69) = 27.215, *p* < 0.001], and a significant task × contrast interaction [*F*(2,69) = 2.933, *p* = 0.05]. There was no effect of contrast (*p* > 0.1). Follow-up Tukey tests revealed that within both contrasts, the F1 distances were larger in the native French productions, smaller distances in the repetition task and the smallest differences in naming task (all differences were significant at *p* < 0.05), note that in native French productions the distances for the assimilated contrast were larger than for the uncategorized contrast, see **Figure 6** for an illustration of the results.

### CORRELATION ANALYSES

In order to examine the relationship between L2 perception and production, Spearman-rank correlation analyses (that are used when the relationship between the two variables is monotonic but not linear) were performed on individual’s mean identification and DS scores for each contrast separately. All correlations were not significant (*p* > 0.1).

### FOLLOW-UP ANALYSES

**FIGURE 7:**
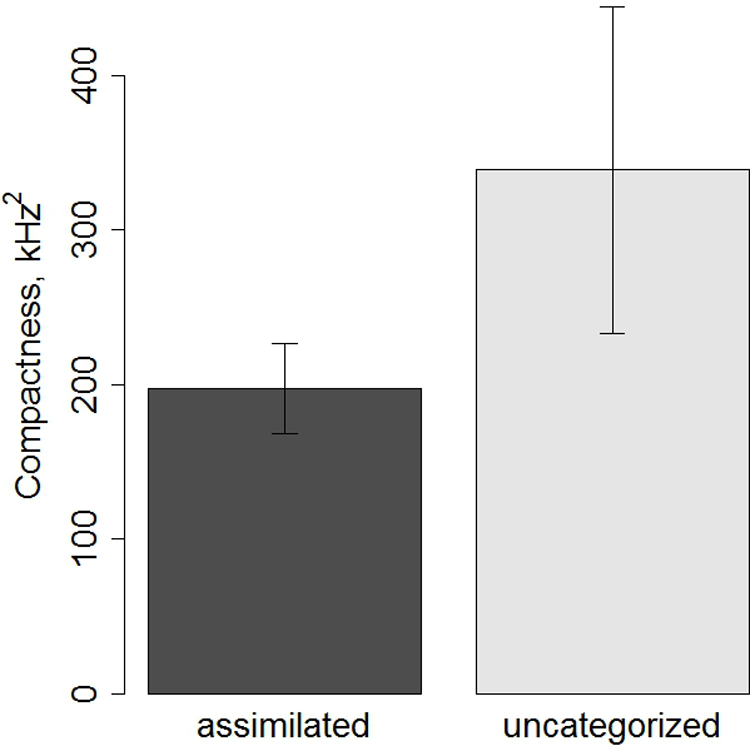
**Compactness of assimilated and uncategorized vowels produced by Spanish speakers in the repetition task.** Mean compactness scores are shown, and error bars represent ±1 standard error of the mean.

**FIGURE 8:**
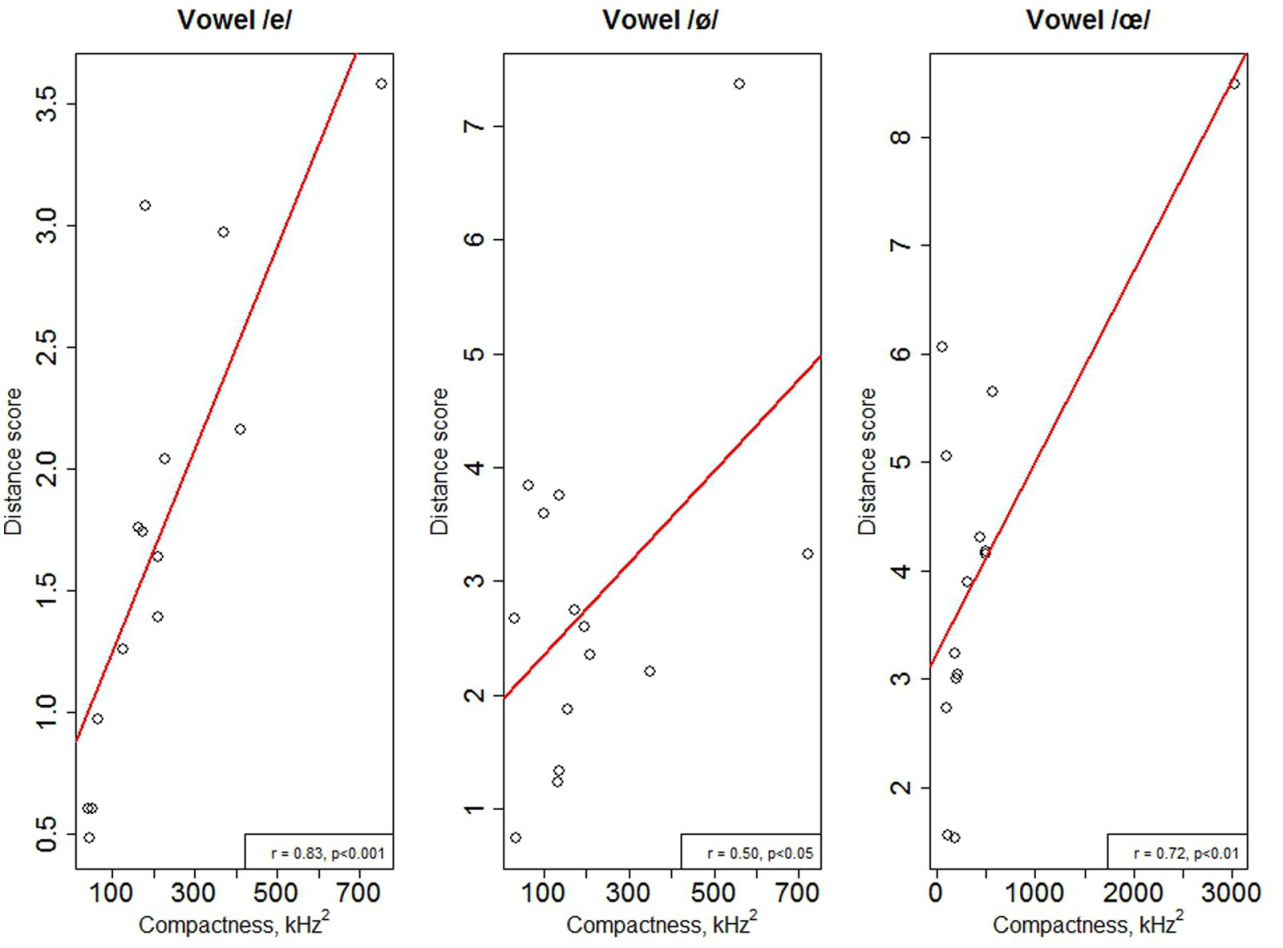
**Scatterplots of the mean individual productions of the vowels /e/, /ø/, and /œ/ and regression lines for the effects of the L2 compactness score (CS_**L2**_) on the production accuracy of these vowels**.

The compactness of L2 productions (CS_L2_) in the repetition task was computed for the /e/, /ε/, /ø/, and /œ/ vowels for each speaker using the same formula as the one used to calculate the compactness of Spanish productions. In the naming task, too few productions for each speaker were available to estimate compactness. Bartlett’s test showed a violation of homogeneity of variances [χ^2^(1) = 35.6, *p* < 0.001]. Kruskal–Wallis rank test was performed on the CS_L2_, with a between-group factor of contrast (assimilated versus uncategorized). There was no effect of contrast on CS_L2_ [χ^2^(1) = 0.78, *p* < 0.37]. However, as can be seen from **Figure 7**, there is a tendency for uncategorized vowels to be produced more variably than the assimilated ones. Nonetheless, the cross-speaker variability is too large for an effect to emerge between the two contrasts. Follow-up tests revealed no effect of vowel for both contrasts (*p* > 0.1). Separate Pearson-correlational analyses were run for each vowel between the CS_L2_ and the DS for the corresponding vowel. The results are summarized in **Table 4**, and the significant correlations are illustrated in the **Figure 8**. The results showed that for the /e/, /ø/, and /œ/ vowels more compact productions were associated with smaller distances between the productions and native target French vowels.

**Table 4:** Results of Pearson-correlation analyses between the DS and CS_**L2**_.

Vowel	Pearson coefficient *r* and *p*-value
/e/	*r* = 0.83, *p* < 0.01
/ε/	*r* = 0.21, ns
/ø/	*r* = 0.50, *p* = 0.03
/œ/	*r* = 0.72, *p* < 0.01

## GENERAL DISCUSSION

Our results will be discussed with reference to **Figure 1**. Firstly, we will consider the L2 perception performance and its relation to L1 phonology (link A). Secondly, we will discuss L2 production performance and examine the relationship between L2 perception and L2 production (link C). Finally, we will discuss the effect of individual differences in the phonetic properties of L1 productions on L2 production (link D).

### L2 PERCEPTION PERFORMANCE

Our results revealed that the assimilated vowels were perceived better than the uncategorized ones (51% and 37.1% correct responses, respectively). In terms of PAM this suggests that the /e/-/ε/ vowels assimilated in SC manner to the Spanish /e/ category and that the /ø/-/œ/ vowels are not discriminated from each other (or from neighboring native categories, see below for details). Participants’ better perception on assimilated than on uncategorized vowels can partially be explained by our use of a larger response set in a 5FCI task rather than the typical two-choice identification or discrimination tasks. Our task makes it possible to evaluate perception not only within a contrast, but also within the larger L2 phonological space. For the assimilated contrast, Spanish speakers only rarely responded outside their contrast (see **Table 3A**), and so their chance level was close to 50%. On the other hand, for the uncategorized contrast shown in **Table 3B**, all five phonemes in the response set were frequently used when identifying the /ø/ and /œ/ vowels, and therefore, their chance level was much lower, perhaps even at about 20%. If we had used the classical two-choice identification task for the uncategorized vowels, we would probably have obtained identification performance of around 50%, as for the assimilated vowels.

The perception performance on the assimilated contrast suggests assimilation in a SC manner (rather than CG) to the Spanish /e/ category. On more than a half of the trials, both the /e/ and /ε/ vowels were identified as the /e/ vowel (59% and 55% of /e/ identifications respectively, see **Table 1**). These results suggest that both L2 vowels were perceived as being poor instances of the SP /e/ category (Flege, 1995; Best and Tyler, 2007).

The low performance on the uncategorized French /ø/-/œ/ contrast (37.1% correct responses) shows that participants have major difficulties in identifying these L2 vowels. **Table 3B** shows that on about 1/3 of the trials each vowel of the contrast was mistaken for the other vowel. This result is consistent with the PAM, according to which the perception of uncategorized L2 contrasts is expected to be poor if the L2 phones are perceived as being very close to each other (Best, 1995; Best and Tyler, 2007). Since the mid-close/mid-open height opposition does not exist in SP, both FR vowels map onto one mid SP dimension (presumably, the Spanish rounded /o/ vowel) and are therefore perceived as being very similar. The poor identification score observed for the mid-close/mid-open /ø/-/œ/ contrast reflects not only the difficulties in perceiving the vowels in the contrast but also in distinguishing them from the back rounded vowels (/o/–/ɔ/). This opposition (i.e., back–front for rounded vowels) does not exist in Spanish. Meunier et al. (2004) have shown that Spanish speakers categorize the French vowels /ø/-/œ/ as the Spanish /o/. Assimilation of French front rounded to back rounded vowels has also been observed for natives of other languages lacking front rounded vowels. AE learners of French, for instance, categorized French /y/-/œ/ vowels as AE back vowels (Levy, 2009). Importantly, the author has shown that assimilation patterns varied as function of consonant context with more AE /^j^u/ and /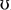/ responses for the French /y/ and /œ/ vowels, respectively, in bilabial context than in alveolar context. In another study, however, AE experienced but not inexperienced learners showed no context effect on discrimination of the French /y/-/u/ and /œ/-/u/ vowel pairs (Levy and Strange, 2008). Our study was designed to avoid contextual effects on the perception performance of Spanish speakers. It remains to be tested whether Spanish speakers’ perception performance is affected by consonant context effects.

In sum, the results of our study suggest that when the perception of L2 French mid-close/mid-open vowel contrasts is assessed using a large L2 phonological response set (i.e., one that is not limited to the tested contrasts), Spanish speakers experience more difficulties with new contrasts than with those that are similar to sounds in the L1 phonological system. Moreover, our results also point to the importance, especially in the case of new sounds, of using larger response sets that make it possible to assess L2 perception outside the phonetic contrast, rather than using two-choice tasks as is typically done.

### L2 PRODUCTION PERFORMANCE

The production of two French vowel contrasts was assessed in repetition and naming tasks. We conducted three different analyses to examine participants’ performance. First, we assessed and compared vowel production accuracy on each task. Here we computed the distance (DS) separating the F1/F2 values of each produced L2 vowel from the target-category acoustic space and we analyzed the distinctness of the contrasting L2 vowels by calculating the F1-difference separating them. Second, we measured the compactness (i.e., inverse of variability) of the acoustic spaces in the production of the four tested /e/-/ε/-/ø/-/œ/ vowels on the repetition task.

We compared the production of the assimilated and the uncategorised contrasts on two tasks, repetition and vowel naming. We expected that Spanish participants’ productions would be more accurate in the repetition task than in the naming task for both types of contrasts. The results revealed no effect of task on the production accuracy of either French contrasts. These results suggest that the acoustico-phonetic and phonological representations that underlie the production of the vowels in the repetition and naming tasks, respectively, are of similar quality/accuracy.

Like for perception, the production of the assimilated contrast was more accurate than that of the uncategorized one. This pattern can be attributed to differences in the number of existing French minimal pairs containing the vowels /e/-/ε/ and /ø/-/œ/ respectively. In French, minimal pairs based on /e/-/ε/ are abundant and contain high frequent words (e.g., mes-mais, tes-tais, ses-sais, gré-grès for “my”-“but,” “your”-“keep silent,” “his”-“I know,” “will”-“sandstone”; Féry, 2003), whereas the only two existent /ø/-/œ/ minimal pairs (i.e., veulent-veule, jeune-jeûne for “they want”-“weak,” “young”-“fast”) are extremely rare (Tranel, 1987 in Durand and Lyche, 2004). The PAM-L2 model predicts that the likelihood of discerning differences between contrasting vowels depends on the “adaptive significance of detecting the difference between minimally contrasting L2 words” (Best and Tyler, 2007, p. 30). Our results suggest that poor production performance on the /ø/-/œ/ contrast is due to (partial) merging of the /ø/-/œ/ French vowels into one mid-height category toward /ø/ vowel. In particular, as it can be seen in **Figure 6**, the /œ/ vowel is produced more /ø/-like with considerably low F1 (more closed) compared to native French speakers’ productions. However, since /ø/-/œ/ lexical contrasts are quasi-inexistent in French, the homophony of /ø/-/œ/ vowels is not a great disadvantage for learners of French.

Difficulties in production of French front rounded vowels can also be due to difficulties in mastering the frontness dimension (F2) for rounded vowels. Levy and Law (2010) found that late AE speakers of French tended to produce French front rounded vowels excessively “back” (i.e., lower F2), as judged by native French speakers. This tendency, however, was not observed in our acoustical analysis of Spanish speakers’ productions of the /ø/-/œ/ vowels. In fact, their productions were actually slightly more front than those of French speakers (see **Figure 6**), thereby supporting the “merging” hypothesis.

The results of the analyses on the distinctness of the L2 vowels within a contrast, that is, on the acoustic distance separating them, revealed an effect of task for both contrasts. The distinctness, as reflected by the height distance (F1) between the contrasting vowels, was larger on the repetition than on the naming task. The results on the distinctness of productions suggest that the acoustico-phonetic representations underlying the production of both assimilated and uncategorized contrasts are more accurate in a repetition task than are the internal phonological representations tapped by the vowel naming task. Nevertheless, even on the repetition task the vowels are not produced as distinctly as they are by native speakers. Vowels in both contrasts were repeated with comparable (no statistical effect of contrast) but limited distinctness. This result suggests that the mid-close/mid-open height distinction that does not exist in Spanish is very difficult for Spanish speakers of French, independently of the perceptual similarity of the L2 contrasts to L1 sounds. Levy and Law assessed production of front rounded vowels by AE highly experienced speakers of French (e.g., a mean of 8 years of formal education and 3.5 years of immersion into a French-speaking country). They concluded that an uncategorized vowel /œ/ that assimilated to several native categories is of particular difficulty even for L2 experienced speakers. Native French speakers judged relatively low AE productions of this vowel and that independently of the consonant context used (Levy and Law, 2010).

The observed differences in the effects of task on the measures of accuracy (repetition = naming) and of distinctness (repetition > naming) could be due to two factors. First, the statistical analyses used to assess the task effects on the production accuracy included both within-subject and between subject variability (i.e., mixed-effects model), whereas they included only between-subject variability for the distinctness measure. There was therefore more variability in the former analysis which could have prevented the effect from emerging. Second, the accuracy measure (i.e., DS) compares the F1 and F2 values of each vowel (i.e., /e/ and /ε/, and /ø/ and /œ/) to those of the corresponding target vowel produced by native French speakers; the distinctness, on the other hand, only reflects the F1-differences in the production of contrasting vowels for each subject. The latter measure therefore allows us to capture small differences in the height dimension that might be masked by using a joint measure including F1 and F2 in the DS measure.

Only the productions in the repetition task were analyzed for their compactness since there were too few productions for each speaker in the naming task to estimate their compactness. Although the statistical analyses did not reveal differences in compactness between two contrasts (most likely due to large cross-speaker variability), it can be seen from **Figure 7** that the productions of uncategorized vowels seem to be less compact than those of assimilated vowels (mean CS of 338 versus 197 kHz^2^ respectively), suggesting that the underlying representations for the former have not yet been stabilized. However, in absence of significant results this interpretation should be taken with caution.

Correlation analyses revealed that compactness was strongly correlated with the L2 production accuracy measure (DS) for three L2 vowels (i.e., /e/, /ø/, and /œ/): speakers whose productions were compact in L2 space were also more accurate. Our recent L2 production training study has shown that successful learning of L2 sounds is accompanied by reduced variability (increased compactness) of the trained sounds (Kartushina et al., submitted). The absence of such a correlation for the French /ε/ vowel that is highly similar to the Spanish /e/ vowel (Kartushina and Frauenfelder, 2013) suggests that L2 Spanish speakers re-use the L1 /e/ category to produce this L2 vowel and have not yet established a separate L2 category. The presence of such a correlation for the remaining three (dissimilar) vowels suggests that L1-Spanish-L2-French speakers are successful at establishing new L2 categories when auditory examples are provided (e.g., during a repetition task).

### RELATIONSHIP BETWEEN L2 PERCEPTION AND L2 PRODUCTION

In examining the relationship between L2 perception and production (link C), we have combined three approaches. First, we looked at group performance by comparing average accuracy on the perception and production of L2 contrasts. Second, we explored individual performance by a) running correlational analyses between L2 perception and production performance across speakers, and b) running by-subject mixed-effects regression analyses to test the effect of L2 perception accuracy on L2 production, taking into account individual variability. Unfortunately, only few studies have compared L2 perception and production performance directly, with even fewer having used the two former approaches (e.g., Flege, 1993; Flege et al., 1999; Peperkamp and Bouchon, 2011) and none having used all three.

The average results for both L2 perception and production tasks revealed an effect of the type of contrast: vowels in the assimilated contrast were perceived and produced better than those in the uncategorized contrast. This result could be taken to suggest that L2 perception and production are related. However, the results of the correlation and of mixed-effects generalized regression analyses revealed no relationship between L2 perception and production; speakers’ identification accuracy did not predict their production accuracy.

This divergence as a function of the type of analysis conducted is neither novel nor unexpected. For example, in a study on the effect of L2 perception training on L2 production, a group effect was observed (i.e., improved perception of the trained vowels led to improved production), but there was no relationship between the improvement in these capacities across individual speakers (Bradlow et al., 1997). This result as well as ours shows that when individual differences in performance are taken into account, a relationship between perception and production is generally no longer observed. Barr et al. (2013) have claimed that such statistical models that include random factors (e.g., cross-participant variability) allow for better generalizability of the results. To our knowledge, our study is the first to include such variability (mixed-effects models) in assessing the relationship between L2 perception and L2 production. It suggests that L2 phonological perception and production have different underlying representations [see Edwards (1995) for a distinction between “input” and “output” representations]. This result is consistent with the growing body of research showing dissociations between L2 perception and production (Neufeld, 1979; Sheldon and Strange, 1982; Kassaian, 2011) and no correlation between these two modalities (Kluge et al., 2007; Hattori and Iverson, 2010; Peperkamp and Bouchon, 2011). Brain imaging studies provide some support for this interpretation by showing a partial dissociation between the brain-structure correlates of L2 phonetic perception and production (Golestani and Pallier, 2007; Golestani et al., 2007).

Other L2 studies have shown that perception and production may be aligned only in proficient L2 speakers (Flege and Schmidt, 1995). Since the speakers tested in our study may be considered as intermediate-level L2 speakers (as reflected by a brief questionnaire filled by the participants at the beginning of the experiment and by French teacher report), with further increases in proficiency, a relationship between perception and production may begin to be observed. However, since our sample is too small, additional empirical data must be collected and analyzed using statistical tools that take individual variability into account in order to draw more definitive conclusions.

### THE ROLE OF INDIVIDUAL PHONETIC L1 PRODUCTION IN L2 PRONUNCIATION

#### L1 compactness

One of the main aims of our study was to evaluate the relation between intra-speaker variability in the production of native sounds and the production accuracy of L2 contrasts (link D in **Figure 1**). Native speakers vary in the way they produce the same L1 sound, not only in terms of the mean acoustic values of the L1 phonemes (e.g., more closed versus more open productions of the /e/ vowel by Spanish speakers, see Kartushina and Frauenfelder, 2013), but also in terms of their variability. Some speakers have less variable realizations (compact acoustic spaces for given L1 sounds), whereas others do not (dispersed acoustic spaces). We hypothesized (in line with the SLM) that speakers whose productions are more compact in L1 space would be more likely to discern the phonetic differences between L1 and L2 sounds and to establish more precise L2 categories, than speakers whose productions are more dispersed.

Two CSs were considered. The first, CS_V_, refers to the compactness of the acoustic space for the Spanish /e/ vowel and was used to predict the production performance on the French /e/ and /ε/ vowels that assimilate to this Spanish vowel. The second, CS_G_, refers to the global compactness score (i.e., global indicator of within-category variability) for the production of all Spanish vowels (/i/, /e/, /a/, /o/, and /u/) and was used to predict L2 production performance on the French /ø/ and /œ/ vowels that have no clearly similar category in L1 space and therefore are uncategorized. The results revealed significant effects of L1 compactness on L2 production accuracy for both assimilated and uncategorized contrasts: (1) Spanish speakers with more compact distributions for the Spanish /e/ vowel (CS_V_) were better at producing the similar French /e/ and /ε/ vowels; (2) Spanish speakers with more compact overall distributions for the five Spanish vowels (CS_G_) were better at producing the uncategorized French /ø/ and /œ/ vowels.

The results for the assimilated vowels corroborate and extend to production our previous findings on the effect of L1 compactness on L2 perception (Kartushina and Frauenfelder, 2013). The previous and present findings taken together suggest that individual native phonetic realizations transfer to the L2 space and affect both the perception (link B) and production (link D) of similar L2 sounds.

The results on the French uncategorized /ø/ and /œ/ vowels revealed that speakers whose vowel productions in L1 space were more compact produced them more accurately than those whose L1 space was more dispersed. In other words, speakers whose Spanish front and back vowels were less variable and mainly restricted acoustically to the front and back positions^6^ (thus leaving the acoustically central positions unoccupied or “blank,” the term proposed by Escudero and Boersma (2004) produced L2 acoustically central vowels better than those whose Spanish front and back vowels extended to the center of the acoustic space. This result suggests that “accurate” L1 speakers are more likely than “sloppy” L1 speakers to create novel categories for the new (i.e., uncategorized) L2 vowels. Other research has shown the effects of cross-speaker and cross-language variability in L1 productions on the perception of native (Perkell et al., 2004) and foreign-sounds respectively (Meunier et al., 2003, 2004). For example, Perkell et al. (2004) have shown that AE speakers who produced the native contrastive vowels /I/ and /i/ more distinctly and compactly discriminated these vowels more accurately than those whose productions were less distinct and overlapped more.

#### Acoustic distance between L1 and L2 sounds

The distance score measure (DS-FR) that estimated the distance between the native Spanish /e/ vowel and the non-native French /e/ and /ε/ vowels showed that the closer the L1 /e/ category is to the target French vowel, the higher the production accuracy for this target vowel is. These results suggest that L2 speakers re-use exemplars of the native /e/ category to produce similar L2 categories. However, it is important to note that the L2 productions are not prototypical L1 exemplars. We computed additional DSs between the Spanish (i.e., /e/) and French (i.e., /e/ and /ε/) productions that confirm this: on average, the L2 /e/ and /ε/ productions were 5 and 4.5 DS units away from the native Spanish /e/ category, respectively. However, since the Spanish vowels produced in the reading task are more prone to co-articulation effects than the French vowels that were produced in isolation, these comparisons should be further confirmed with acoustic analyses in which both L1 and L2 vowels are recorded in similar phonetic environments. It should be noted however, that we measured the F1/F2 of the Spanish vowels produced in the reading task at the mid portion of the vowel in order to reduce such coarticulation effects.

One of the SLM’s claims is that bilinguals strive to maintain a contrast between L1 categories and similar L2 categories in a common interphonological space. Although our participants were not fluent Spanish-French bilinguals but were quite advanced L2-French learners, they nevertheless exhibited a tendency to maintain the contrast between the Spanish /e/ and the similar French /e/ and /ε/ vowels on both tasks. The ellipses representing 1 standard deviation distributions of the vowels (i.e., vowel acoustic space) tend to not overlap between the native /e/ and the similar /e/ and /ε/ L2 categories. Moreover, even in the naming task (when no auditory example is given), participants tend to use realizations that are different from those of the L1 /e/ vowel in producing the similar L2 sounds: their L2 /e/ and /ε/ productions are more closed and opened respectively. This result taken together with that of the DS-FR suggests that Spanish speakers re-use non-prototypical L1 tokens to produce similar L2 vowels, and that they try to maintain the differences between the L1 sounds and similar L2 sounds.

The significant interaction between the compactness of the L1 /e/ category and its distance from the target French vowels suggests that the effect of compactness on production accuracy is stronger when the acoustic distance between the Spanish and French sounds is smaller. This can be due to the fact that when such distances are smaller, greater compactness is necessary in order to still retain some “blank space” around the native category in order to ensure accurate production of a newly formed L2 category/sound.

### LIMITATIONS AND DIRECTIONS FOR FUTURE RESEARCH

In this section we propose some improvements for studying L2 production both in terms of the tasks and measures used to evaluate its accuracy. We used two different production tasks, naming and repetition, in order to tap into phonological and acoustico-phonetic representations, respectively. A similar methodological approach could be taken for the perception tasks as well. The 5FCI task that we used to assess L2 perception is well suited for evaluating internal L2 phonological representations. However, it cannot provide much information about the detailed properties of the phonetic representations underlying the perception of L2 vowels. A categorization task (using continua) that more finely reveals the categorical perception (i.e., category boundaries) of L2 sounds could be included to assess phonetic processing. By using tasks that reflect the perception and production of L2 sounds at different representational levels (e.g., acoustic, phonological, lexical), we could draw more solid conclusions about the relationship between the two capacities at comparable representational levels.

In computing the global compactness of L1 acoustic space, we added together the specific compactness of the five Spanish vowels, and inferred that those speakers who had more compact vowels (i.e., little within-category variability) were likely to have larger “empty” acoustic spaces. Another approach would be to assess the size of the “empty” spaces more exactly by subtracting the CS of the five Spanish vowels from the “total” space (i.e., the area of a triangle delimited by the extreme vowels /i/, /a/, and /u/). Alternatively, we could assess the ratio between the between-category and the within-category variability, as suggested by the editor of this issue. Also, other properties of individuals’ L1 productions could be predictive of L2 production and perception performance. These include the distribution of vowel categories in acoustic space (e.g., the extension of the categories in openness and height dimensions, i.e., from the lowest F1 and F2 to the highest F1 and F2 respectively), and their distinctness in production (e.g., whether they overlap with each other or not).

Last, we used compactness measures to evaluate the variability of productions in L1 space. These measures partially depend upon the articulatory skill of the speakers: the more skilled and precise the speakers, the more compact their productions. If our results are indeed attributable to articulatory skill, then we would expect speakers who are “articulatorily precise” in L1 to also be precise in L2. However, additional correlational analyses did not reveal any relationship between the compactness in the L1 and L2 productions. This result, however, should be taken with caution since compactness was assessed on productions using different tasks in L1 and L2. Alternatively, individual differences in compactness can be due to factors other than articulatory skill. Bosch and Ramon-Casas (2011) assessed the variability (i.e., compactness) of productions and their accuracy in simultaneous and early bilinguals. They found that the productions of simultaneous bilinguals were more variable and less accurate (i.e., more mispronunciations: producing /e/ in words involving the /ε/ vowel, and vice versa) than that of early bilinguals. The authors interpreted the more variable vowel productions together with greater vowel selection errors in the simultaneous bilinguals as reflecting less stable phonological representations. This study did not, however, report data on individual differences in the variability of the productions. More research is needed to understand the underlying causes of differences in compactness (i.e., variability) across speakers and its relationship to L2 production and perception in terms of the underlying causes – differences in articulation skill, and/or in the quality of phonological representations, or even in other factors.

## CONCLUSION

Our study has addressed three questions. First, what is the role of the similarity between L2 sounds and L1 categories in L2 identification? Second, what is the relationship between the perception and production of non-native (L2) sounds? And third, what is the role of individual, native (L1) productions in determining L2 pronunciation accuracy?

Native Spanish speakers learning French in high school were assessed on their perception and production of two French mid-close/mid-open contrasts, (1) /e/-/ε/, that perceptually assimilates to one Spanish /e/ category; and (2) /ø/-/œ/, that is dissimilar to any existing Spanish category (i.e., an “uncategorized” contrast). Native Spanish productions were also recorded, and two phonetic measures were computed for each individual: the variability in the production of these native vowels (represented by the compactness of vowel categories in F1/F2 acoustic space), and the position of the vowels in the acoustic space. Productions of French vowels by native French speakers were also recorded, and compared to productions of these French vowels by the Spanish speakers.

We found, first, that the assimilated contrast /e/-/ε/ was identified better than the uncategorized one /ø/-/œ/. This result is at odds with the SLM which predicts that new sounds are acquired better than similar sounds. It supports the PAM-2 that predicts poor performance on uncategorized contrasts if L2 phones are perceived as being very close to each other and to the same L1 sounds.

With regard to the second question, the group results revealed that the poorly identified contrast was generally produced poorly. However, the results of the analyses that took into account individual variability in the production and perception of L2 sounds revealed no relationship between these two modalities. This goes against the traditionally held view that L2 production depends upon L2 perception, and is in line with the growing body of research showing at least partial dissociations between L2 perception and production, and between their underlying neural mechanisms.

Finally, we have shown that the phonetic properties of an individual’s L1 productions (i.e., acoustic compactness of L1 categories, and their position in the individual’s L1 space) predicted L2 production accuracy. These results are consistent with the claim by SLM of a shared inter-phonological space between languages, since they show that L1 and L2 sounds are related to each other at the acoustico-phonetic level, within individuals. Our results are in line with surface transfer hypothesis and suggest a transfer of individual phonetic categories, in addition to more abstract, phonological ones to L2 production. Other studies have also pointed to the role of L1 phonetic properties in L2 perception. For example, Hallé et al. (1999, p. 301, 303) showed that French speakers tend to hear AE /r/ as /w/-like although, from the phonological point of view, they should have categorized it as French /r/. The authors conclude that “detailed phonetic properties contribute substantially more than abstract phonological characterizations alone to non-native speech perception” and suggest that “a realistic phonology should incorporate phonetic-articulatory descriptions of the segmental categories in each language”. Similarly, Bohn and Best (2012) showed that cross-language phonetic similarity predicts L2 perception better than phonological correspondences.

Our results provide evidence in favor of the role of individual phonetic properties of native productions in predicting L2 production accuracy, but also point to the need for further investigation of individual-specific factors affecting L2 production. Together with our previously published findings showing that L1 production also influences L2 perception, these results highlight the malleability of phonetic processing, and demonstrate that pre-existing features of the native space in production partly determine how new elements can be accommodated in that space.

## Conflict of Interest Statement

The authors declare that the research was conducted in the absence of any commercial or financial relationships that could be construed as a potential conflict of interest.

## Footnotes

1 It is important to mention that the relationships presented in this figure are not exclusive neither sufficient in understanding L2 acquisition phenomena. Only for the sake of clarity, other links and bidirectional arrows were removed.

2 Note that the difference between language-specific versus individual-specific L1 categories in **Figure 1** can be captured by de Saussure’s distinction between “language” (abstract language system) and “parole” (concrete language use by individuals; Hjelmslev, 1942).

3 Note that 20 vowels (two exemplars per vowel) produced by one (i.e., the sixth) speaker were only used for the familiarization phase in the identification task.

4 The identification performance on the four vowels of interest only is reported in this study.

5 The use of different speakers during testing and familiarization phases was done in order to make sure that the perception performance during the testing phase was not biased by learning that might have occurred in the familiarization phase.

6 Note, that there were no speakers whose productions were superimposed and/or collapsed in the central region.
